# Plasma biomarkers and genetics in the diagnosis and prediction of Alzheimer’s disease

**DOI:** 10.1093/brain/awac128

**Published:** 2022-04-06

**Authors:** Joshua Stevenson-Hoare, Amanda Heslegrave, Ganna Leonenko, Dina Fathalla, Eftychia Bellou, Lauren Luckcuck, Rachel Marshall, Rebecca Sims, Bryan Paul Morgan, John Hardy, Bart de Strooper, Julie Williams, Henrik Zetterberg, Valentina Escott-Price

**Affiliations:** Dementia Research Institute, Cardiff University, Cardiff, UK; Dementia Research Institute, University College London, London, UK; Department of Neurodegenerative Disease, UCL Institute of Neurology, Queen Square, London, UK; Dementia Research Institute, Cardiff University, Cardiff, UK; Dementia Research Institute, Cardiff University, Cardiff, UK; Dementia Research Institute, Cardiff University, Cardiff, UK; Dementia Research Institute, Cardiff University, Cardiff, UK; Division of Neuroscience and Mental Health, Cardiff University, Cardiff, UK; Division of Neuroscience and Mental Health, Cardiff University, Cardiff, UK; Dementia Research Institute, Cardiff University, Cardiff, UK; Dementia Research Institute, University College London, London, UK; Department of Neurodegenerative Disease, UCL Institute of Neurology, Queen Square, London, UK; Dementia Research Institute, University College London, London, UK; VIB Center for Brain and Disease Research, 3000 Leuven, Belgium; KU Leuven, Leuven Brain Institute, 3000 Leuven, Belgium; Dementia Research Institute, Cardiff University, Cardiff, UK; Dementia Research Institute, University College London, London, UK; Department of Neurodegenerative Disease, UCL Institute of Neurology, Queen Square, London, UK; Hong Kong Center for Neurodegenerative Diseases, Hong Kong, China; Department of Psychiatry and Neurochemistry, Institute of Neuroscience and Physiology, the Sahlgrenska Academy at the University of Gothenburg, Mölndal, Sweden; Clinical Neurochemistry Laboratory, Sahlgrenska University Hospital, Mölndal, Sweden; Dementia Research Institute, Cardiff University, Cardiff, UK; Division of Neuroscience and Mental Health, Cardiff University, Cardiff, UK

**Keywords:** Plasma biomarkers, genome-wide association study, Alzheimer’s disease

## Abstract

Plasma biomarkers for Alzheimer’s disease-related pathologies have undergone rapid developments during the past few years, and there are now well-validated blood tests for amyloid and tau pathology, as well as neurodegeneration and astrocytic activation. To define Alzheimer’s disease with biomarkers rather than clinical assessment, we assessed prediction of research-diagnosed disease status using these biomarkers and tested genetic variants associated with the biomarkers that may reflect more accurately the risk of biochemically defined Alzheimer’s disease instead of the risk of dementia.

In a cohort of Alzheimer’s disease cases [*n* = 1439, mean age 68 years (standard deviation = 8.2)] and screened controls [*n* = 508, mean age 82 years (standard deviation = 6.8)], we measured plasma concentrations of the 40 and 42 amino acid-long amyloid-β (Aβ) fragments (Aβ_40_ and Aβ_42_, respectively), tau phosphorylated at amino acid 181 (P-tau181), neurofilament light (NfL) and glial fibrillary acidic protein (GFAP) using state-of-the-art Single molecule array (Simoa) technology. We tested the relationships between the biomarkers and Alzheimer’s disease genetic risk, age at onset and disease duration. We also conducted a genome-wide association study for association of disease risk genes with these biomarkers.

The prediction accuracy of Alzheimer’s disease clinical diagnosis by the combination of all biomarkers, *APOE* and polygenic risk score reached area under receiver operating characteristic curve (AUC) = 0.81, with the most significant contributors being ε4, Aβ_40_ or Aβ_42_, GFAP and NfL. All biomarkers were significantly associated with age in cases and controls (*P* < 4.3 × 10^−5^). Concentrations of the Aβ-related biomarkers in plasma were significantly lower in cases compared with controls, whereas other biomarker levels were significantly higher in cases.

In the case-control genome-wide analyses, *APOE*-ε4 was associated with all biomarkers (*P* = 0.011−4.78 × 10^−8^), except NfL. No novel genome-wide significant single nucleotide polymorphisms were found in the case-control design; however, in a case-only analysis, we found two independent genome-wide significant associations between the Aβ_42_/Aβ_40_ ratio and *WWOX* and *COPG2* genes.

Disease prediction modelling by the combination of all biomarkers indicates that the variance attributed to P-tau181 is mostly captured by *APOE*-ε4, whereas Aβ_40_, Aβ_42_, GFAP and NfL biomarkers explain additional variation over and above *APOE*. We identified novel plausible genome wide-significant genes associated with Aβ_42_/Aβ_40_ ratio in a sample which is 50 times smaller than current genome-wide association studies in Alzheimer’s disease.

## Teaser

Stevenson-Hoare *et al.* show that five blood plasma biomarkers can together be used to diagnose and predict the onset of Alzheimer’s disease. Levels of the biomarkers are associated with age in both cases and controls, and show strong associations with genes including APOE-ε4.

## Introduction

Alzheimer’s disease (AD) is one of the greatest health challenges, affecting tens of millions of people worldwide. The clinical diagnosis of this disease is, however, often inaccurate; around 25% of people with clinical AD do not have underlying pathology at autopsy, and many people who have not yet developed AD-type dementia have incipient pathology, the prevalence of which increases with age.^1^ Detecting AD at the earliest possible stage remains essential to combating its effects and to further our understanding of this devastating illness. By diagnosing early, we can better understand how the disease progresses, plan and implement treatments earlier and monitor response to drugs currently being trialled.

Amyloid-β (Aβ) and tau pathology are the defining pathological features of AD.^2^ For many years, it has been possible to detect AD pathology (amyloid aggregation, tau tangles and neurodegeneration) using imaging and CSF biomarkers. Although CSF and PET biomarkers of Aβ and tau are highly accurate for detecting disease pathology,^3^ the costs, invasive nature and low availability of the tools needed to detect these biomarkers hamper their feasibility for use in clinical diagnostic practice and for screening in clinical trials.

Assays for plasma Aβ fragments [ratio of Aβ_1–42_ (Aβ_42_) to Aβ_1–40_ (Aβ_40_)] reflect brain amyloidosis^4–7^; however, these assays have limitations, including the impact of substantial peripheral Aβ production.^8^ By contrast, CSF and plasma tau phosphorylated at threonine 181 (P-tau181) is a highly specific pathological marker of AD that remains normal in other dementias.^9,10^ Glial fibrillary acidic protein (GFAP) and neurofilament light chain (NfL) are putative non-amyloid plasma-based biomarkers indicative of ongoing neuroinflammatory and neurodegenerative disease processes. Increased GFAP suggests abnormal activation and proliferation of astrocytes, for instance secondary to neuronal damage. It has been shown that GFAP levels in plasma and CSF are higher in AD and correlate with cognitive impairment.^11–13^ Plasma NfL is a marker of neuronal injury, increased in AD,^14^ but this biomarker has low specificity, because increases are also reported in several other neurodegenerative disorders.^13,15,16^ Thus, while NfL has potential as a monitoring biomarker, GFAP might be a valuable prognostic biomarker, predicting incident dementia.^13^ Recent reports show that plasma P-tau181 concentration starts to increase around 15 years prior to clinical disease onset in familial AD^17^ and that plasma P-tau181 predicts disease neuropathology at least 8 years prior to autopsy in sporadic disease.^10^

Early disease prediction can be helped with genetic data as an individual’s genetic makeup does not change over time and genetic data are precise and inexpensive to measure; however, the prediction accuracy using genetics is limited.^18^ Biomarkers, in contrast to genetics, can only indicate the presence of AD pathology after the disease has already been triggered, i.e. a biomarker change marks the onset of a pathological process. Nevertheless, the prediction accuracy of e.g. P-tau181 and P-tau217 for discriminating AD from other neurodegenerative diseases,^19–21^ when combined with *APOE* genotype, memory and executive function phenotypes, was reported to reach area under receiver operating characteristic curve (AUC) > 90% in predicting the progression from mild cognitive impairment (MCI) to AD in two relatively small samples of participants (*n* = 340 and 543).^22^

Identifying genetic loci associated with biomarkers could aid understanding of the specific pathophysiological components underpinning these biomarkers. Genome-wide association studies (GWAS) of CSF biomarkers in AD case/control samples have found loci in genes *GEMC1* and *OSTN*^23^ as well as more commonly reported loci such as the *TREM* cluster, *APOE*, *APOC* and *TOMM40.*^24^ However, these have also only focused on small sets of biomarkers, typically P-tau181 and Aβ_42_. GWAS of blood plasma P-tau181 and NfL levels^25,26^ have identified only loci within the *APOE* genomic region, and only for P-tau181. Investigation of the relationship between AD polygenic risk score (PRS) and plasma P-tau181^27^ has revealed highly significant associations with PRS containing the *APOE* region (*P* = 3 × 10^−18^−7 × 10^−15^) and moderate association when *APOE* was excluded. GWAS studies for plasma Aβ_40_, Aβ_42_ and Aβ_42/40_ ratio in non-demented participants from population-based studies have identified GWAS significant variants in *APOE* and *BACE1* genes, and *APP*, *PSEN2*, *CCK* and *ZNF397* genes in gene-based analysis.^28^

The aims of this study were (i) to test the prediction ability of the biomarkers for clinical AD diagnosis in our cohort (over and above commonly used predictors such as *APOE*, age and AD PRS); and (ii) to identify genetic loci associated with these plasma biomarkers. The latter may shed light on which single nucleotide polymorphisms (SNPs) associated with clinical AD are also associated with plasma biomarkers. This could help to further refine the relevance of the AD GWAS genes to different biological processes, which the biomarkers represent. To that end, we measured plasma biomarkers in a sample of 1439 early and late onset AD cases {mean age 68 years [standard deviation (SD) = 8.0]} and 508 elderly screened controls [mean age 82 years (SD = 6.7)]. We used ultrasensitive Single molecule array (Simoa) assays to measure P-tau181, NfL, GFAP, Aβ_40_ and Aβ_42_, and calculated the ratio of Aβ_42/40_. We then tested these biomarkers for association with the clinical diagnosis of AD and, in case samples, the relationship of the biomarkers with age at sample collection, age at onset and disease duration. To identify genetic loci associated with these biomarkers, we undertook a GWAS for P-tau181, NfL, Aβ_40_, Aβ_42_, ratio of Aβ_42/40_ and GFAP biomarkers in the largest case-control sample set to date.

## Materials and methods

###  

#### Alzheimer’s Disease Cardiff Cohort

The Alzheimer’s Disease Cardiff Cohort (ADCC) was collected between 2004 and 2020 using MRC, Moondance Foundation and Health and Care Research Wales (HCRW) funding. The cohort collection used a standardized clinical and comprehensive neuropsychological assessment (validated by Holmes *et al.*^29^), see more details in Section 1 of the Supplementary material. AD diagnosis was not supported by any biochemical or imaging measures (e.g. CSF or PET) due to the funds allocated to the study collecting the data.

We used plasma samples collected from 1439 early and late onset sporadic AD cases and 508 screened elderly controls. Information on age at assessment, sex, *APOE* genotype and genome-wide array genotyping was available for all 1947 samples. Within cases, information was also available for *n* = 1319 individuals on age at onset, and duration of disease was calculated for these samples. Details of the sample demographics are in Table 1.

**Table 1:** Summary of demographics and plasma biomarker summary characteristics in ADCC, post-outlier removal

	Controls (*n* = 508)	Cases (*n* = 1439)
**Demographics**		
Age, years	82.2 (6.72)	68.1 (8.03)
Sex, male/female	221/287	748/691
Age at onset	N/A	62.4 (7.9)
Duration, years	N/A	5.3 (3.6)
**Biomarkers**		
Aβ_40_	140 (40.0)	94.5 (34.4)
Aβ_42_	7.50 (2.05)	5.00 (1.84)
GFAP	196 (85.3)	215 (103)
NfL	32.9 (13.7)	31.0 (13.9)
P-tau181	3.18 (1.54)	4.10 (1.90)
Aβ_42_/Aβ_40_	0.0556 (0.013)	0.0543 (0.014)

Values are mean (SD). Biomarker values are in μg/ml.

### Biomarkers

Biomarkers were tested for 1986 individual plasma samples from the ADCC. P-tau181 concentration was measured using the Simoa P-tau181 Advantage Kit, whilst Aβ_40_, Aβ_42_, NfL and GFAP concentrations were measured using the Simoa Human Neurology 4-Plex E (N4PE) assay (Quanterix). The measurements were performed in one round of experiments using one batch of reagents with the analysts blinded to diagnosis and clinical data. All measurements for all five analytes were above the limit of detection of the assays. Intra-assay coefficients of variation were below 10%. These data were then matched to phenotype information. Thirty-nine samples were removed at this stage based on missing/mismatching data for age and gender or due to ID duplication, leaving 1947 individuals for further analysis. Samples were excluded for each biomarker analysis on a case-by-case basis, based on outlier thresholds calculated using Median Absolute Deviation (MAD).^30^ This method is more robust to remote outliers than the mean and SD method and copes better with skewed data due to its reliance on non-parametric measures of central tendency and variation. Pearson’s correlations between biomarkers were calculated for the 1735 samples which had no outlier measurements for any biomarker. Details of biomarker distributions are in Table 1.

### Genetics

Individuals for this analysis were included if both genetic and biomarker information were available, totalling 1947 individuals in the final dataset. All individuals had information available on *APOE* genotype (ɛ2ɛ2 = 8, ɛ2ɛ3 = 145, ɛ2ɛ4 = 33, ɛ3ɛ3 = 844, ɛ3ɛ4 = 620 and ɛ4ɛ4 = 239). Quality control (QC) of the genetic data was performed for cases and controls together, the QC steps used are reported elsewhere^31,32^ and in Section 2 of the Supplementary material. Genotyped data were aligned to human genome assembly GRCh37/hg19 and imputed via Michigan Imputation server using Minimac3^33^ with the Haplotype Reference Consortium (HRC)^34^ reference panel. Post-imputation QC used thresholds of minor allele frequency (MAF) < 5%, poor accuracy of imputation (INFO) < 0.8, MISS > 5% and Hardy–Weinberg equilibrium *P* ≤ 10^−6^. This resulted in a final dataset containing 4 618 496 variants.

### Statistical analysis

The association of biomarkers with age at onset and disease duration in cases, and with age at interview in cases and in controls (separately), was tested with linear regression where the biomarker was the outcome variable, controlling for sex. For all following analyses the biomarkers were adjusted for age and standardized to have a mean of zero and standard deviation of one. The correlations between the biomarkers were assessed with Pearson’s correlation.

The association of AD case/control status by the biomarkers was tested using logistic regression, accounting for sex, *APOE* and PRS without the *APOE* region (chromosome 19:44.4–46.5 Mb) using the glm() function in R. The most parsimonious model was derived with the backwards stepwise approach [step() function in R]. The prediction accuracy was assessed by means of the AUC, using the auc() function in R.

The *APOE* region was represented by the number of ɛ2 and ɛ4 alleles which we used as two predictor variables. The PRS without *APOE* region (PRSnoAPOE) was used to account for the remaining genetic effect. For the PRS calculation we used the summary statistics from the largest *clinically assessed* late-onset case-control GWAS study on AD available at the time of analysis (*n* = 63 926)^35^. PRS were generated with the PLINK genetic data analysis toolset^36^ for *P*-value threshold *P* ≤ 0.1 on LD-clumped SNPs by retaining the SNP with the smallest *P*-value excluding variants with r^2^ > 0.1 in a 1000 kb window, see details in.^37^ Prior to analyses PRSnoAPOE was adjusted for five principal components and then standardised.

All statistical analyses were performed in R-statistical software (https://www.R-project.org/). The plots were generated using the *ggplot2* package with custom scripts generated in house.

The results of the biomarkers’ association with the clinical/demographic characteristics are presented without correction for multiple testing, since these analyses are hypothesis-driven.

### Genetic analysis

SNP-based association analyses were performed for each biomarker using linear regression model with PLINK. Association analyses of SNPs with the biomarkers were adjusted for age and sex, five principal components (PCs) and case-control status (‘caseness’). The adjustment for caseness was introduced to reduce the variation due to potential differences in association pattern of biomarkers between cases and controls, whilst using all available samples to maintain the statistical power. In addition, association analyses for cases and controls were also conducted separately. Since the *APOE* region is not well covered by the Illumina arrays used to genotype the ADCC dataset, we tested association of the biomarkers with the number of directly genotyped *APOE*-ɛ4 alleles. PCs were computed using PLINK and the number of PCs was determined via visual inspection of the pairwise PC scatter plots. The GWAS significance level was set to the commonly accepted *P* < 5 × 10^−8^. We did not further adjust this for the six biomarkers as the biomarker levels were measured in the same sample and are not independent.

To investigate further the variants of interest, we used Combined Annotation-Dependent Depletion (CADD) and RegulomeDB (RDB) scores for SNPs accessible within the Functional Mapping and Annotation of Genetic Associations (FUMA) on-line tool.^38^ CADD is a tool for scoring the deleteriousness of single nucleotide variants as well as insertion/deletions variants in the human genome.^39,40^ RDB^41^ is a categorical score from 1a to 7, representing regulatory functionality of SNPs based on expression quantitative trait loci (eQTLs) and chromatin marks. 1a is the highest score, indicating that the SNP has the most biological evidence to be a regulatory element.

We compared our GWAS biomarker association results to AD genome-wide significant findings,^35^ assessing all SNPs in the ADCC GWAS within ±20 kB of the GWAS-significant SNPs. The replication significance level was set to nominal significance level *P* < 0.05.

To summarize the association results from all variants in a gene, accounting for number of variants and linkage disequilibrium (LD) between them, we used Multi-marker Analysis of GenoMic Annotation (MAGMA, v1.09b).^42^ For the gene-based analysis, we mapped a SNP to a gene (as defined by NCBI 37.3) if it resided within the gene boundaries. The LD between SNPs was estimated with the European reference panel in 1000 Genomes phase 3. The significance level for the gene-based analysis results was set to the commonly accepted *P* < 2.5 × 10^−6^.

For the pathway analyses, 10 271 gene sets were downloaded from Reactome, Biocarta, KEGG and Pathway Interaction Databases.^32^ The pathway analyses were performed using the ‘competitive’ option in MAGMA, assessing whether the genes in a gene set are more strongly associated with the phenotype than in other gene sets in the genome. We adopted the false discovery rate (FDR ≤ 0.05) approach [p.adjust() function in R with method = ‘fdr’] to correct for multiple testing the results of the pathway analyses.

### Data availability

GWAS summary statistics for the top results (*P* ≤ 1 × 10^−5^) are listed in the main text of the paper and Supplementary material. Full GWAS summary statistics are available from the authors upon request.

## Results

### Biomarker results in relation to Alzheimer’s disease, age at onset and disease duration

**Figure 1:**
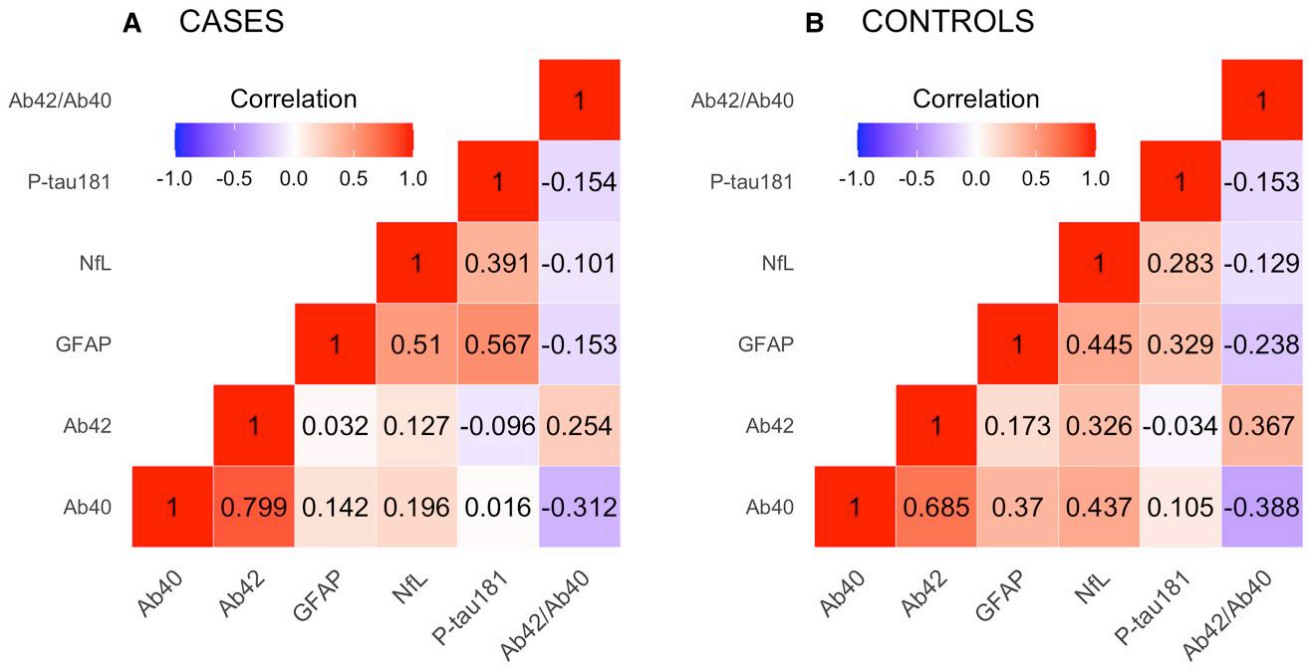
**Pearson correlation between biomarkers in cases and controls.** (**A**) Cases and (**B**) controls.

The correlation pattern between the biomarkers was similar for cases and controls and agreed with the results reportedby Cullen *et al*.^43^ The correlation between Aβ_42_ and Aβ_40_ values was high (*r* = 0.8 in cases and 0.7 in controls, *P* < 10^−16^). The lowest correlation was observed between P-tau181 and Aβ-related biomarkers, see Fig. 1.

To assess whether the disease stage is captured by the biomarkers, we explored the relationship between biomarkers, age of onset and disease duration in cases. Table 2 summarizes the results. In this case-only analysis, age at onset was strongly positively associated with Aβ_40_, Aβ_42_, GFAP and NfL (*P*-values ≤ 4.2 × 10^−23^), moderately with P-tau181 (*P* = 0.0023) and negatively with Aβ_42_/Aβ_40_ (*P* = 4.8 × 10^−4^). The biomarkers GFAP, NfL and P-tau181 show significant increase in females as compared to males (*P* = 9.0 × 10^−23^, 1.4 × 10^−7^ and 2.1 × 10^−8^, respectively). This in part replicates the finding in Kumar-Singh *et al*.,^44^ who showed that age-of-onset of *PSEN1*-linked familial AD correlated negatively with Aβ_42_/Aβ_40_ but positively with Aβ_40_ levels. Longer disease duration was strongly associated with elevated levels of GFAP and NfL (*P* = 2.9 × 10^−6^and 1.2 × 10^−12^, respectively) and moderately associated with increase of Aβ_40_ and P-tau181 levels (*P* = 0.027 and 0.008, respectively).

**Table 2:** Beta coefficients, SE and *P*-values for linear regressions predicting biomarkers from age at onset and disease duration in AD cases, controlling for age and sex

	*n*	Age at onset			Duration		
		B	SE	*P*	B	SE	*P*
Aβ_40_	1219	0.042	0.003	1.9 × 10^−35^	0.016	0.007	0.027
Aβ_42_	1219	0.034	0.003	4.2 × 10^−23^	0.013	0.007	0.077
GFAP	1301	0.034	0.003	7.1 × 10^−24^	0.034	0.007	2.9 × 10^−6^
NfL	1275	0.048	0.003	1.1 × 10^−44^	0.050	0.007	1.2 × 10^−12^
pTau-181	1309	0.011	0.003	0.0023	0.020	0.008	0.008
Aβ_42_/Aβ_40_	1215	−0.012	0.003	0.0005	−0.004	0.008	0.592

In controls, all biomarkers were positively associated with age at interview (*P*-value ranked between 1.2 × 10^−7^ for Aβ_42_ and 1.9 × 10^−30^ for NfL) and negatively with the ratio Aβ_42_/Aβ_40_ (*P* = 1.2 × 10^−10^) (see Table 3), indicating that all biomarkers are sensitive to age and will show less discrimination between AD cases and controls if AD cases with earlier onset (∼65–68 years) are compared with elderly screened controls (Supplementary Fig. 1).

**Table 3:** Beta coefficients, SE and *P*-values for linear regressions predicting biomarkers from age at interview in cases and controls, controlling for sex

	Cases (max *n* = 1439)				Controls (max *n* = 508)			
	*n*	B	SE	*P*	*n*	B	SE	*P*
Aβ_40_	1415	0.041	0.003	2.9 × 10^−37^	492	0.064	0.006	1.2 × 10^−22^
Aβ_42_	1417	0.034	0.003	4.6 × 10^−25^	486	0.036	0.007	1.2 × 10^−7^
GFAP	1394	0.034	0.003	8.4 × 10^−28^	501	0.052	0.006	4.6 × 10^−16^
NfL	1361	0.051	0.003	1.2 × 10^−54^	478	0.074	0.006	1.9 × 10^−30^
P-tau181	1389	0.014	0.003	4.3 × 10^−5^	472	0.038	0.007	3.5 × 10^−08^
Aβ_42_/Aβ_40_	1413	−0.010	0.003	0.0018	481	−0.044	0.007	1.2 × 10^−10^

Next, we assessed the prediction accuracy of disease status in our sample. The prediction accuracy of the case-control status by sex and *APOE* genotype resulted in AUC = 0.74 and R^2^ = 0.21. All biomarkers were significantly associated with AD status when tested separately (Table 4 and Supplementary Fig. 2). The prediction accuracies, however, were moderate with the highest prediction accuracy AUC = 0.66 and 0.65 for Aβ_42_ and P-tau181, respectively.

**Table 4:** Results of logistic regressions predicting AD status from each biomarker, adjusted for age and sex

	B	SE	*P*	R^2^	AUC
Aβ_40_	−0.44	0.058	3.5 × 10^−14^	0.05	0.63
Aβ_42_	−0.56	0.059	2.8 × 10^−21^	0.08	0.66
GFAP	0.55	0.067	2.4 × 10^−16^	0.07	0.64
NfL	0.47	0.066	1.1 × 10^−12^	0.05	0.63
P-tau181	0.55	0.067	1.4 × 10^−16^	0.07	0.65
Aβ_42_/Aβ_40_	−0.18	0.055	0.0009	0.01	0.56

Data include 1302 cases and 421 controls after excluding the missing values list-wise.

The prediction accuracy of a model combining all biomarkers and genetics (*APOE*-ɛ4, *APOE*-ɛ2, PRS without *APOE* region) was AUC = 0.81, R^2^ = 0.29. The most parsimonious model that predicted the outcome with the same accuracy as above (derived using stepwise regression) included all predictors except Aβ_42_ and P-tau181 (*APOE*-ɛ4 B = 1.3, *P* = 2.02 × 10^−24^; *APOE*-ɛ2 B = −0.45, *P* = 0.011; PRSnoAPOE B = 0.14, *P* = 0.033; Aβ_40_ B = −0.62, *P* = 6.6 × 10^−18^; GFAP, B = 0.29, *P* = 3.9 × 10^−4^; NfL B = 0.45, *P* = 4.6 × 10^−8^; Aβ_42_/Aβ_40_ B = −0.20, *P* = 0.003).

This model highlights the importance of all genetic predictors and the Aβ_40_, GFAP and NfL biomarkers. The variance of Aβ_42_ was captured by Aβ_40_, as the correlation between these biomarkers was high. Indeed, when Aβ_40_ was dropped from the model, then Aβ_42_ became a significant predictor (B = −0.59, *P* = 9.6 × 10^−12^). In both models, the ratio of Aβ_42_/Aβ_40_ was significant, but it changed its direction of effect depending on which marker was included (B = 0.20, *P* = 0.005 and B = −0.20, *P* = 0.003, when Aβ_42_ or Aβ_40_ was included, respectively) P-tau181 was dropped from the model by the stepwise regression, however this should not be interpreted as P-tau181 being fully explained by the genetic predictors. In a model with only P-tau181 and genetics (APOE-ɛ4, APOE-ɛ2, PRSnoAPOE), P-tau181 remained highly significant over and above genetics (B = 0.38, *P* = 4.5 × 10^−8^).

The model with all biomarkers but without genetic predictors had an accuracy of AUC = 0.75 and explained variance of R^2^ = 0.18. In this model, the same biomarkers as above showed significant association, with the addition of the P-tau181 biomarker (B = 0.18, *P* = 0.022), indicating that the P-tau181 signal may be explained by genetics, whereas the other significant biomarkers (Aβ-related, GFAP and NfL) add to the prediction over and above genetics.

### Genome-wide association study

We performed three sets of GWAS (cases only, controls only, all samples) in ADCC with the five biomarkers (Aβ_40_, Aβ_42_, NfL, P-tau181, GFAP) and the Aβ_42_/Aβ_40_ ratio as outcome measures. The top SNPs with an association *P*-value ≤ 1 × 10^−5^ are presented in Supplementary Tables 1–6. In the case-control analysis, *APOE*-ɛ4 was associated with all biomarkers (*P* = 0.011−4.78 × 10^−8^; Supplementary Tables 1, 2 and 4–6), except NfL (Supplementary Table 3).

We compared the GWAS we performed for biomarkers to the genome-wide significant SNPs from a large clinically assessed AD GWAS study^35^ (see Supplementary Table 7). The strongest associations for the GWAS index *APOE* SNP (rs429358) were for P-tau181 and GFAP (*P* = 0.001 and 0.002, respectively; Supplementary Table 7). Interestingly, SNPs in or near the *WWOX* gene were at least nominally associated with all biomarkers. The strongest association was found for GFAP (*P* = 1.2 × 10^−5^) for a SNP situated 2.7 kb away from the GWAS index *WWOX* SNP.

**Figure 2:**
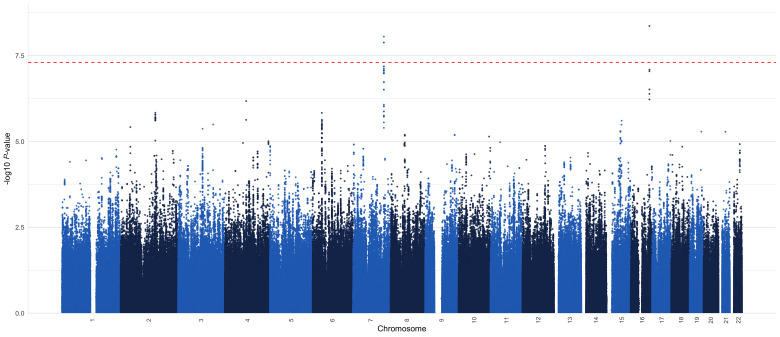
Aβ _42_ /Aβ _40_ case-only GWAS (*n* = 1420 cases).

The GWAS of the five biomarkers and the Aβ_42_/Aβ_40_ ratio in controls only and in all samples did not reveal any genome-wide significant loci. In the cases only GWAS, however, we observed two genome-wide significant loci for the Aβ_42_/Aβ_40_ ratio (Supplementary Table 6 and Fig. 2). The lead SNPs for these loci lie within the intronic region of their respective genes (*COPG2* and *WWOX*), with the WWOX variant predicted to function as an enhancer.

**Figure 3:**
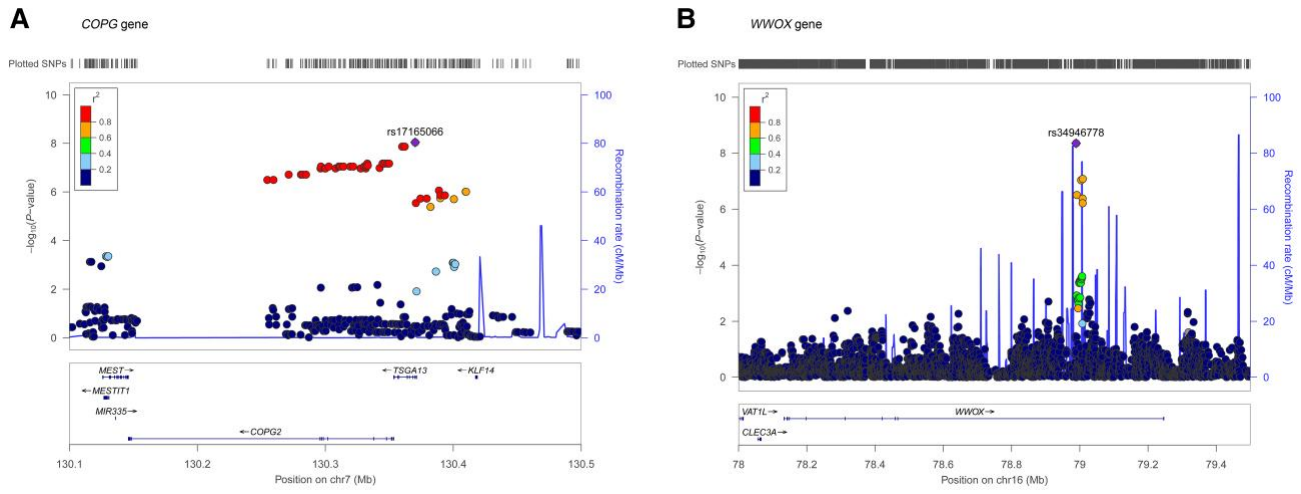
**Genome-wide significant regions associated with Aβ_42_/Aβ_40_ in case-only analysis (*n* = 1420 cases).** (**A**) *COPG* and (**B**) *WWOX*.

The first genome-wide significant locus was a high LD region on chromosome 7 spanning from 130.2–130.4 Mb and covering genes *COPG2* (chr7:130 146 080–130 353 598) and *TSGA13* (chr7:130 353 486–130 371 406) with the lead SNP rs17165066 [chr7:130 370 267, B = 0.15, standard error (SE) = 0.026, *P* = 8.9 × 10^−9^]. This SNP tags 50 other SNPs with r^2^ > 0.8; see Manhattan plot (Fig. 2) and LocusZoom plot (Fig. 3A). Moreover, this region contains two SNPs (rs10264429 and rs375839317, MAF = 0.06, 0.07, respectively), which are in high LD with the lead SNP (r^2^ = 0.84 and 0.71, respectively) and have CADD scores = 13.6, 12.48, which are greater than the suggestive threshold for a SNP to be deleterious (CADD > 12.37). The rs77696591 (MAF = 0.06) intergenic variant is also tagged by the lead SNP (r^2^ = 0.87) and has an RDB score = 3a, i.e. has ‘putatively functional impact on gene regulation’. The lead SNP rs17165066 was not statistically significant in the clinically assessed AD GWAS.^35^

The second genome-wide significant region was on chromosome 16 in the *WWOX* gene (chr16:78 133 327–79 246 564), which has also been linked to AD by GWAS.^35^ The lead SNP rs34946778 (chr16:78989116, B = 0.15, SE = 0.026, *P* = 4.36 × 10^−9^) was not statistically significant in the AD GWAS.^35^ The linkage disequilibrium was r^2^ = 0.0014 between the AD GWAS lead SNP (rs62039712) and the SNP identified in our study (rs34946778).

Finally, the number of *APOE*-ɛ4 alleles was associated with Aβ_40_ (B = −0.072, *P* = 1.1 × 10^−2^), Aβ_42_ (B = −0.015, *P* = 6.3 × 10^−7^), Aβ_42_/Aβ_40_ (B = −0.15, *P* = 1.05 × 10^−5^), GFAP (B = 0.1, *P* = 1.3 × 10^−3^) and P-tau181 (B = 0.18, *P* = 4.7 × 10^−8^) but not with NfL (*P* = 0.40).

## Discussion

We demonstrated that the prediction accuracy for AD status by the combination of blood biomarkers, sex, *APOE* and PRS reaches AUC = 0.81 (R^2^ = 0.29) with the most significant contributors being *APOE-*ɛ4, Aβ_40_ and GFAP. This AUC value is lower than that reported in Palmqvist *et al*.,^22^ likely due to our controls being systematically older than cases, with the diagnostic accuracies for AD being decreased with age.^45^ Note that Aβ_42_ becomes a highly significant predictor when Aβ_40_ is dropped from the model and vice versa, although a stepwise regression recommended dropping Aβ_42_ over Aβ_40_. The prediction accuracy by all biomarkers without genetic predictors was AUC = 0.75, which is slightly higher than the accuracy by genetic predictors alone (AUC = 0.73 in our sample). Interestingly, P-tau181 was not significant if genetic predictors were included in the model and became significant only when no genetic predictors were used, indicating that genetic factors, *APOE*-ɛ4 in particular, influence plasma P-tau181 levels. However, an advantage of P-tau181 as a biomarker over other predictors (e.g. genetics) is that it is a relatively inexpensive blood biomarker and does not reveal any sensitive genetic information.

In controls, age at interview was positively associated with all biomarkers (*P*-value ranged between 1.2 × 10^−7^ for Aβ_42_ and 1.9 × 10^−30^ for NfL) and negatively associated with the ratio Aβ_42_/Aβ_40_ (*P* = 1.2 × 10^−10^), indicating that all biomarkers are sensitive to age or pre-clinical age-related neurodegenerative pathologies.

In case-only analyses, age at onset was significantly associated with all biomarkers, in particular, positively with Aβ_40_, Aβ_42_, GFAP, NfL and P-tau181 and negatively with the ratio Aβ_42_/Aβ_40_. In addition to age at onset, GFAP, NfL and P-tau181 were also associated with the disease duration, with similar effect sizes indicating that the associations can be attributed to age in general, rather than to a particular feature of the disease development and progression. These findings are in line with other recent studies. Chatterjee *et al*.^46^ demonstrate that plasma GFAP levels are elevated in cognitively normal older adults at risk of AD. Aschenbrenner *et al.*^47^ conclude that NfL can be used to monitor both cognitive decline due to normal ageing and dementia. Lantero Rodriguez *et al.*^10^ report that the main increase in plasma P-tau181 occurred between 8 and 4 years prior to death in patients with AD neuropathology, whereas patients without pathology and controls exhibited minor, although significant, increases in P-tau181 up until death.

The Aβ_40_ and Aβ_42_ results showing increasing concentration with age in both cases and controls support the earlier finding that Aβ_40_ and Aβ_42_ levels are increased before the onset of sporadic AD.^48–50^ It has also been shown that the biomarker distributions are more similar between subjects with and without AD in elderly subjects than in young subjects.^45^ When comparing cases and controls in our sample, we found that cases have lower concentrations of Aβ_40_ and Aβ_42_ in plasma, accounting for age. This might indicate that cases, despite early onset, are in the advanced stage of the disease [mean disease duration 5.3 years (SE = 3.6)] in our sample). An earlier study^50^ showed that Aβ_40_ and Aβ_42_ levels are elevated in some patients before and during the early stages of AD but decline thereafter. Our results show similar association patterns (lower Aβ_42_/Aβ_40_ is associated with increased age) to the recent report^7^ for participants of all ages and diagnoses who were enrolled in a longitudinal study of memory and ageing. Another study,^5^ which included cognitively normal individuals, patients with mild cognitive impairment and patients with AD, found no significant correlations between the biomarker values and age. A population-based study^51^ reports results in a cohort where all individuals were born in the same week, but blood samples were collected within the testing period of 2.6 years. Within this very limited age range, Aβ_42_ (but not Aβ_40_) was significantly positively associated with age. Therefore Aβ_42_/Aβ_40_ was also positively associated with age. In our study with a much wider age range, both Aβ_42_ and Aβ_40_ were significantly positively associated with age. The ratio Aβ_42_/Aβ_40_ was negatively associated with age because the increase in Aβ_40_ was greater than that in Aβ_42_ (Table 4 and Supplementary Fig. 2). To summarize the Aβ data, the biomarker is sensitive to age and potentially other clinical conditions and phenotypes unmeasured and unaccounted for in our and others’ reports. Given this, interpretation of Aβ measurements in the absence of other clinical information is uncertain at best.

In addition, the biomarkers measuring Aβ_40_, Aβ_42_ and P-tau181 levels also have complex trajectories as the disease develops, and this is all in the context of 80% AD diagnostic accuracy. Counterintuitively, it seems that P-tau181 is largely a plaque amyloid marker^52^: it does not go up in progressive supranuclear palsy, it goes up in amyloid mice after onset of plaque pathology^53^ (although it may also increase in tau-overexpressing mice^54^). Aβ, however, goes down when plaque deposition starts and *APOE* correlates with plaque number in a dose-dependent manner.^55^ Thus, *APOE* and P-tau181 correlate positively, because they both largely mark amyloid deposition. When P-tau181 increases, Aβ_42_/Aβ_40_ decreases, because Aβ_42_ sticks to the amyloid plaques, preventing it from leaking into plasma or CSF. An advantage of using the Aβ_42_/Aβ_40_ ratio over the individual biomarkers is that the ratio normalizes high versus low Aβ producers to each other and is a more reliable qualitative test for Aβ status in the brain than Aβ_42_ alone.

We found two independent genome-wide significant associations with the ratio of Aβ_42_/Aβ_40_ in the *COPG2* and *WWOX* genes in a case-only analysis (the lead SNPs in controls were not-significant). In the analysis, which included both cases and controls, these SNPs were not genome-wide significant despite the increased sample size compared to cases-only. The GWAS SNPs found in cases were not statistically significant in controls and had effect sizes in the opposite direction. This may indicate that there are genetic-protein associations that can only be identified when looking at disease-relevant groups (AD in this case).

COPG2 is a part of the coat protein complex I (COPI) which is responsible for retrograde transport from Golgi-to-endoplasmic reticulum. Genetic modulation of the COPI complex leads to changes in amyloid precursor protein processing and a decrease in the amyloid plaque burden in an AD mouse model.^56^

The WW domain-containing oxidoreductase gene (*WWOX*) maps to the ch16q23.1–23.2 region and encodes a 414-amino acid protein composed of two WW domains in its N-terminus and a central short-chain dehydrogenase/reductase domain.^57^ In recent years, abundant evidence from multiple studies has causally linked *WWOX* loss of function with various central nervous system pathologies. *WWOX* dysfunction induced sequential aggregation of tau and Aβ, and caused apoptosis.^58^ The role of *WWOX/WOX1* in AD pathology and in cell death signalling has previously been reported,^59^ as has its role in brain development and pathology.^60^

In conclusion, our results demonstrate that the currently available plasma biomarkers reflect different aspects of AD, some of which can be attributed to ageing in addition to the disease-specific features, while others are specifically related to disease progression mechanisms. Our study shows that biomarker-based diagnosis is not perfect because the biomarker measurements in older controls are similar to those in younger clinically diagnosed AD cases (which likely represents increased prevalence of pre-clinical Alzheimer’s changes in older controls). Biomarkers, however, have the advantage of specificity over clinical assessments, which may confuse dementia subtypes due to phenotypic similarities. Therefore, blood plasma biomarkers can only be a useful tool for the assessment and prediction of AD in the context of other genetic and/or clinical information. The idea that biomarkers alone might provide more accurate prediction for AD remains to be fully validated. Longitudinal studies which use a combination of genetics, plasma biomarkers, brain imaging, and pathology confirmation to differentiate cases and controls could provide accurate analyses moving away from prediction of dementia towards prediction of AD.

## Supplementary Material

awac128_Supplementary_Data
