# Supplementary material for: Plasma biomarkers and genetics in the diagnosis and prediction of Alzheimer’s disease
Source: Brain. 2022 Apr 6;146(2):690–9. doi: 10.1093/brain/awac128 (PMC9924904; doi:10.1093/brain/awac128)

Supplementary Material

Supplementary Section 1

We assessed the likelihood of pseudo-dementia by measuring consistency between clinical scales and obtaining follow-up assessments on all patients. Together with detailed family ascertainment for research diagnosis, our ascertainment protocol recognised the particular challenges in the diagnosis and assessment of EOAD patients including: i) distinguishing patients with anxiety/depression from AD (“pseudo-dementia”); ii) diagnosing behavioural variant frontotemporal dementia (FTD), progressive non-fluent aphasia and semantic dementia and distinguishing these conditions from AD; iii) defining AD variants such as the biparietal syndrome, and iv) providing care and support for patients and families with or at risk of Mendelian neurogenetic disease. Blood samples were collected via venepuncture.

Supplementary Section 2

Genotyping of ADCC dataset was done in different stages and funded by MRC and CADR grant applications. Individuals were included in the analysis that had both genetic and biomarker information available. 107 samples were genotyped as part of the GERAD dataset on the Illumina550 array, with Quality-Control (QC) analysis described in previously (Harrold, 2009) and imputed via Michigan Imputation server using Minimac3 (Das et al., 2016) and Haplotype Reference Consortium (HRC)[ref] reference panel. 1779 samples were genotyped on Illumina GSA array platform from which 1432 individuals were part of EADB project [ref]. The later data was combined and QCed with heterozygosity abs(F-het)>5%, missing data proportion per person >5%, related individuals with $\hat{\pi}$>0.2, gender mismatch, or were population outliers based on European population from 1000 Genomes [The 1000 Genomes Project Consortium. A global reference for human genetic variation. *Nature* **526**, 68–74 (2015)]. Variants were excluded with minor allele frequency (MAF) <1%, missing data proportion (MISS) >5% and Hardy-Weinberg Equilibrium (HWE) ($p \leq 10^{-6}$). Additionally, to exclude batch effect association test was run between controls and variants were excluded with p-value <0.001, retaining 480,021 variants. Genetic data were aligned to human genome assembly GRCh37/hg19 and imputed in the same way as described above. Finally, all data were combined, related individuals were removed with $\hat{\pi}$>0.2, variants were removed with MAF<5%, poor accuracy of imputation

(INFO) <0.8, MISS>5%, and HWE p≤10^{-6}. This resulted in a final dataset containing 4,618,496 variants.

Supplementary Figure 1. Distribution of biomarkers with age at interview in cases and controls. Regression lines plotted with 95% confidence intervals. A) Aβ40, B) Aβ42, C) GFAP, D) NfL, E) P-tau181, F) Aβ42/Aβ40 ratio.

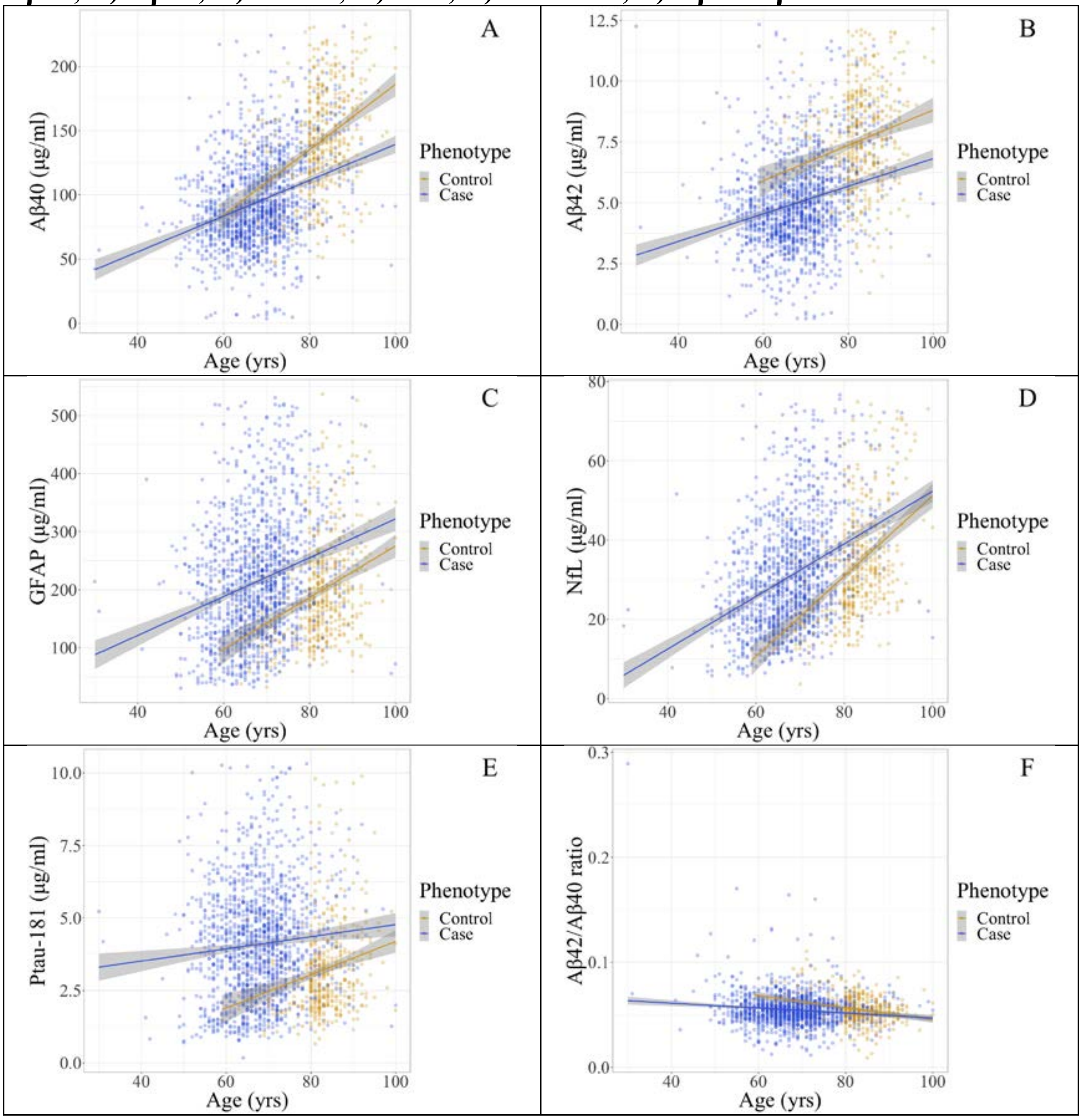

Supplementary Figure 2. Boxplots of biomarkers plotted by AD status. A) Aβ40, B) Aβ42, C) GFAP, D) NfL, E) P-tau181, F) Aβ42/Aβ40 ratio.

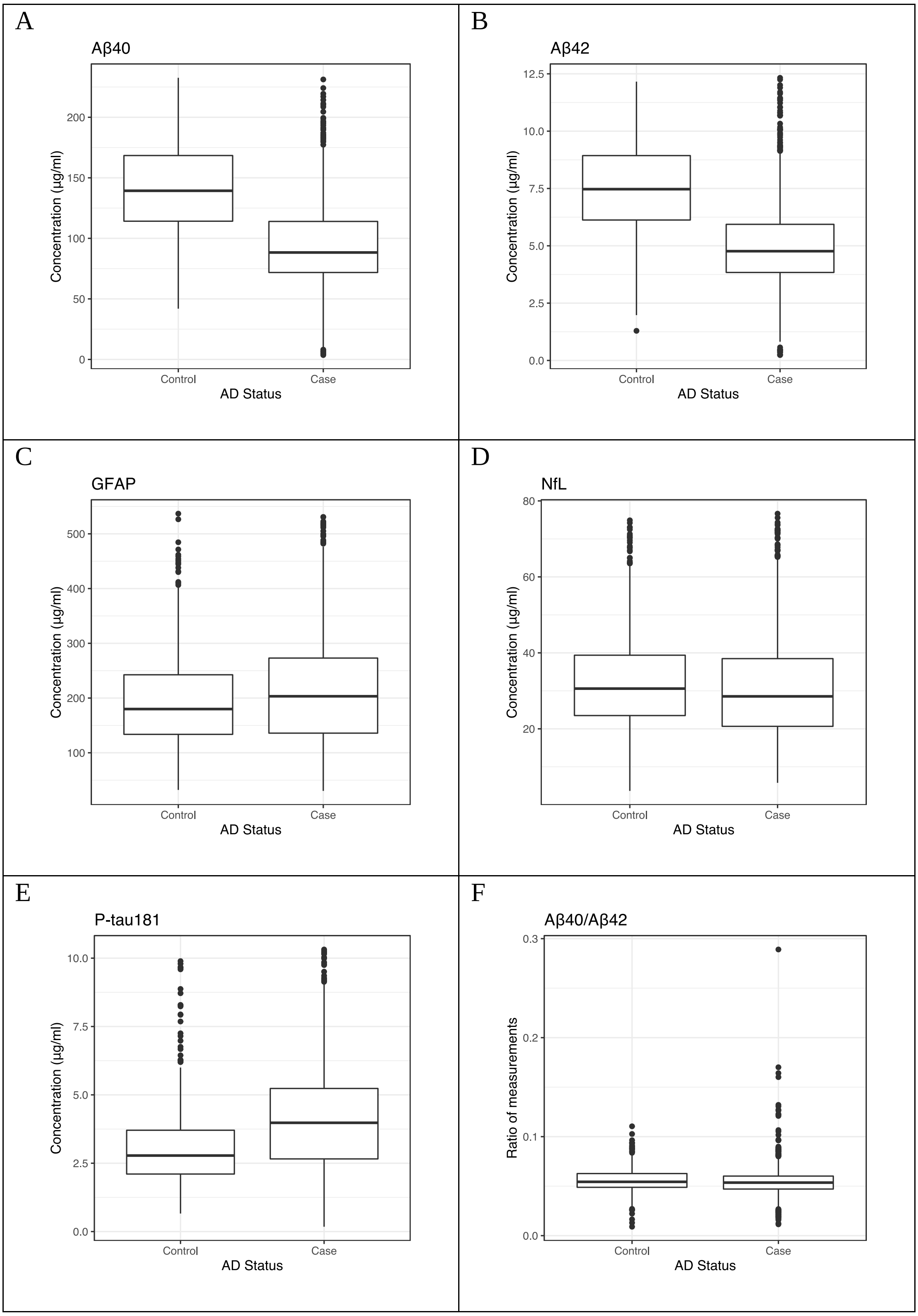

Supplement: awac128_Supplementary_Data [file awac128_Supplementary_Data.zip › brain-2021-02385-File009.pdf]
